# Low oxygen tension reverses antineoplastic effect of iron chelator deferasirox in human glioblastoma cells

**DOI:** 10.1186/s12885-016-2074-y

**Published:** 2016-02-01

**Authors:** Claire Legendre, Sylvie Avril, Catherine Guillet, Emmanuel Garcion

**Affiliations:** INSERM U1066, Micro et Nanomédecines Biomimétiques, IBS - CHU, 4 Rue Larrey, F-49933 Angers, France; PACeM : Plate-forme d’Analyses Cellulaire et Moléculaire, IBS - CHU, 4 Rue Larrey, F-49933 Angers, France

**Keywords:** Glioblastoma, Resistance to therapy, Iron chelation, Deferasirox, Hypoxia

## Abstract

**Background:**

Overcoming resistance to treatment is an essential issue in many cancers including glioblastoma (GBM), the deadliest primary tumor of the central nervous system. As dependence on iron is a key feature of tumor cells, using chelators to reduce iron represents an opportunity to improve conventional GBM therapies. The aim of the present study was, therefore, to investigate the cytostatic and cytotoxic impact of the new iron chelator deferasirox (DFX) on human GBM cells in well-defined clinical situations represented by radiation therapy and mild-hypoxia.

**Results:**

Under experimental normoxic condition (21 % O_2_), deferasirox (DFX) used at 10 μM for 3 days reduced proliferation, led cell cycle arrest in S and G2-M phases and induced cytotoxicity and apoptosis in U251 and U87 GBM cells. The abolition of the antineoplastic DFX effects when cells were co-treated with ferric ammonium sulfate supports the hypothesis that its effects result from its ability to chelate iron. As radiotherapy is the main treatment for GBM, the combination of DFX and X-ray beam irradiation was also investigated. Irradiation at a dose of 16 Gy repressed proliferation, cytotoxicity and apoptosis, but only in U251 cells, while no synergy with DFX was observed in either cell line. Importantly, when the same experiment was conducted in mild-hypoxic conditions (3 % O_2_), the antiproliferative and cytotoxic effects of DFX were abolished, and its ability to deplete iron was also impaired.

**Conclusions:**

Taken together, these in vitro results could raise the question of the benefit of using iron chelators in their native forms under the hypoxic conditions often encountered in solid tumors such as GBM. Developing new chemistry or a new drug delivery system that would keep DFX active in hypoxic cells may be the next step toward their application.

**Electronic supplementary material:**

The online version of this article (doi:10.1186/s12885-016-2074-y) contains supplementary material, which is available to authorized users.

## Background

Glioblastomas (GBM), also called grade IV astrocytomas, are very aggressive primary tumors of the central nervous system. Despite an increase the incidence of up to seven new cases per 100,000 habitants per year, therapeutic approaches have not really evolved in the last 30 years and remain palliative. Treatment generally consists of surgical resection when possible, followed by a combination of external beam radiotherapy with concomitant administration of the orally active alkylating agent temozolomide (TMZ). Hence, the prognosis of GBM is still very poor with a median survival of 14.6 months with radiotherapy amended with TMZ versus 12.1 months with radiotherapy alone [1].

To deal with this negative clinical situation, it is important to find breakthrough therapeutic alternatives while continuing the development of new adjuvant treatments to improve conventional therapy for GBM. Depleting iron levels is a promising approach for GBM. The anticancer activities of iron depletion are based on the fact that neoplastic cells require more iron than normal cells for proliferation [2]. As such, ribonucleotide reductase, which is involved in DNA synthesis and which contains a differic iron site, requires iron as a cofactor to support its activity [2].

In line with this theory, applying iron chelators that bind very tightly to iron thereby promoting its excretion and subsequent depletion in biological systems should be of major interest [3]. Iron chelation therapy has already had a significant clinical impact on diseases other than cancer, primarily to treat iron-overload diseases but also to treat oxidative stress in neurodegenerative diseases [3, 4].

For many years, the most widely used iron chelator was the high affinity constant hexadentate ligand desferrioxamine (DFO). More recently, significant efforts have been made to find new chelators with improved pharmacokinetic and pharmacologic properties, among which the most notable are deferasirox and deferiprone, now available clinically [5]. The anticancer properties of DFO and deferiprone have been tested particularly for brain tumors. Studies conducted at the end of the 1980s in neuroblastoma cells [6, 7] and in children with neuroblastoma [8, 9] showed that DFO has strong antiproliferative and antineoplastic effects. Deferiprone, a bidentate iron chelator, has been shown to have antiproliferative and cytotoxicity activities in neuroblastoma cell lines [7]. However, in vivo, deferiprone fails to reduce tumor growth in the mice xenograft model of human neuroblastoma [10]. In 2005, deferasirox was approved by the FDA for oral route applications thanks to its high iron chelating ability, since when its antineoplastic properties have been tested in numerous human cancer cells and in preclinical studies [11] (Table 1) but never in the context of GBM.

**Table 1:** Protocol based on Deferasirox in cancer therapy

Type of cancer	Mode of action	Ref
Leukemia	CALM-AF10 leukemia cells are susceptible to the cytotoxic effects of DFX (5 μM). However, oral chelation induced by DFX (i.p. 33 mg/kg/day) is not tolerable to leukemic mice and resulted in shortened overall survival.	[32]
	DFX (10 to 60 μM) shows antiproliferative activity as well as cytotoxicity toward several myeloma cells (RPMI 8226, U266 and NCIH929). Mechanisms involved are induced autophagy and repression of mTOR signaling.	[33]
	DFX (20–30 mg/kg/day) synergizes with vitamin D to promote monocyte differentiation and to increase overall survival in elderly patients (≥65 years) with acute myeloid leukemia.	[34]
	DFX (12.5 to 100 μM) reduces viability of murine leukemic cells (EL4 and L1210) and induces apoptosis. Mice bearing L1210 leukemic cells show longer survival than other groups when treated with DFX (p.o. 20 mg/kg/day) with a tumor size smaller.	[35]
	Iron chelation therapy with DFX induces complete remission in a patient with chemotherapy-resistant acute monocytic leukemia	[36]
	DFX (5 to 50 μM) induces apoptosis in myeloid leukemia cells by targeting caspase.	[37]
	DFX (50 μM) induces apoptosis and inhibits NFKB activity in K562 leukemia cells independently of iron deprivation.	[38]
	DFX (17 to 50 μM) inhibits proliferation in human myeloid leukemia cell lines (K562, U937, and HL60). Molecular mechanism responsible for antiproliferative effects involved REDD1/mTOR pathway.	[39]
Esophageal adeno-carcinoma (OAC)	Iron has been shown to potentiate tumorigenesis in OAC but OAC has traditionally been associated with iron deficiency anemia. However, patients with OAC could be considered as candidates for a clinical trial of iron chelation therapy.	[40]
	DFX (10 to 40 μM) reduces cellular viability and proliferation of esophageal tumor cell lines (OE33, OE19 and 0E21) and is able to overcome cisplatin resistance. In human xenograft models, DFX (p.o. 20 mg/kg/day) is able to suppress tumor growth, which was associated with decreased tumor iron levels.	[41]
Lymphoma	DFX (8 to 32 μM) exhibits antitumoral activity against mantle cell lymphoma (HBL-2, Granta-519, Jeko-1). DFX induces apoptosis through caspase-3 activation, down-regulates cyclin D1 and inhibits its related signals, which leads to a G1-S cell cycle arrest.	[42]
	DFX (20 to 100 μM) has dose-dependent cytotoxic effects on human malignant lymphoma cell lines (NCI H28:N78, Ramos, and Jiyoye) with increased sub-G1 portion and apoptosis.	[43]
Lung Cancer	DFX (10 μM) has antiproliferative effect against DMS-53 lung cancer cells and inhibits DMS-53 xenograft growth in nude mice (p.o. 20 mg/kg/day). Mechanisms involved are increased expression of NDRG1 and CIP1/WAF1 and decreased cyclin D1 levels.	[44]
Colorectal cancer	DFX (50 μM) inhibits Wnt signaling in colorectal cancer cells (SW480 and DLD-1) and represses cell proliferation in parallel of the induction of an iron chelation gene signature.	[45]
Liver cancer	DFX (10 to 100 μM) represses proliferation of human hepatocarcinoma cells (HepaRG).	[46]
	In rat (FAO) and human (HUH7) hepatoma cell lines, DFX (10 to 100 μM) decreases cell viability, DNA replication and the number of the cells in G2-M phase and induces apoptosis. Moreover, DFX inhibits polyamine biosynthesis.	[47]
	DFX (10 to 100 μM) induces a cell cycle blockade in G0–G1, decreases cell viability, inhibits DNA replication and induces DNA fragmentation in the human hepatoma cell line HUH7. Importantly, a higher concentration of DFX is necessary to induce cytotoxicity in primary human hepatocyte cultures.	[48]

*i.v.* intravenously, *i.p.* intraperitoneally, *p.o.* per os, *mTOR* mammalian target of rapamycin, *NDRG1* N-myc downstream-regulated gene 1, *CIP1/WAF1* cyclin-dependent kinase inhibitor p21, *NFKB* Nuclear factor-kappaB

The aim of the work was thus to investigate and decipher in vitro the biological effect of the new oral tridentate iron chelator deferasirox (DFX) on two human glioblastoma cell lines, U87 and U251 cells, in terms of proliferation, cell cycle, cytotoxicity and apoptosis. Analyses were performed in conjunction (or not) with external beam radiation treatment and in two oxygenation conditions: experimental normoxia (21 % of oxygen) and brain tumor pathophysiological mild-hypoxia (3 % of oxygen) [12].

## Methods

### Chemicals

All reagents were obtained from Sigma Aldrich (Saint-Quentin Fallavie, France), unless stated otherwise.

### Cell culture

Glioblastoma U87-MG cells (ATCC® HTB-14™) were purchased from the American type Culture Collection (ATCC, LGC Standards, Molsheim, France). U251-MG cells were a gift from C. Griguer and were originally obtained from Dr. D.D. Bigner (Duke University, Durham, NC). U251 and U87 cells are routinely cultured in Dulbecco’s modified Eagle’s medium (DMEM) containing 4.5 g/L glucose and L-glutamine (Lonza, Verviers, Belgium) supplemented with 10 % (v/v) fetal bovine serum (FBS) (Lonza, Verviers, Belgium) and a combination of 100 units/ml penicillin and 100 μg/ml streptomycin. Cells were maintained at 37 °C in a humidified 5 % CO_2_ atmosphere with 21 % or 3 % of oxygen obtained by N_2_ supplementation.

### DFX treatment

Cells were seeded at 15,000 cells/cm^2^. Medium was removed and 24 h after splitting was replaced by DMEM medium with antibiotics and with N1 supplement. Deferasirox (Euromedex, Mundolsheim, France) was suspended in DMSO at a concentration of 0.1 M and used at a final concentration of 10 μM in the cell culture medium for 3 days.

### Irradiation procedure

Cells were seeded at 15,000 cells**/**cm^2^. Medium was removed and replaced 24 h after splitting by DMEM medium with antibiotics and with N1 supplement. Irradiation was performed with the CP-160 cabinet x-ray system (Faxitron, Edimex, Le Plessis Grammoire, Angers, France) which delivers a dose of 1.5 Gy a minute. Irradiation was continued for 10.66 min in order to reach a dose of 16 Gy. The cells were covered during irradiation.

### Proliferation assay

Three days after DFX treatment or irradiation or both, glioblastoma cells were washed with PBS 1× and fixed in 95 % ethanol / 5 % acetic acid (v/v) for 20 min at 4 °C. Hoechst 33342 used at 1.5 μg/mL in PBS 1× was incubated for 30 min. For each condition, 10 fields were counted using a fluorescent microscope (Axiovert 40 CFL Zeiss, Marly le Roi, France) and the number of nuclei were determined.

### Cytotoxicity assay

Three days after DFX treatment or irradiation or both, the release of lactate dehydrogenase (LDH) into cell culture supernatants was measured using a LDH cytotoxicity detection kit (Roche Diagnostics, Meylan, France) according to the manufacturer’s instructions. Glioblastoma cells treated with Triton X-100 at 0.1 % (v/v) were used as positive control of cytotoxicity and assigned the arbitrary value of 100 %.

### Caspase 3 activity

Three days after DFX treatment or irradiation or both, total proteins were isolated from glioblastoma cells by sonication in a lysis buffer (20 mM PIPES pH 7.2, 100 mM NaCl, 1 mM EDTA, 0.1 % KCl w/v, 10 % sucrose w/v, DTT 10 mM and PMSF 100 μM). Proteins (30 μg) were incubated at 37 °C with 80 μM N-acetyl-Asp-Glu-Val-Asp-7-amino-4-methylcoumarin (N-acetyl-DEVD-AMC) and the kinetics of caspase activity was measured with a Fluoroskan Ascent FL (Thermofisher scientific, Illkirch, France) at the excitation/emission wavelength pair of 380/440 nm.

### Cell cycle analysis

Cells from three biological replicates were collected, washed in PBS and fixed in 70 % cold ethanol. Fixed cells were washed twice in PBS and incubated in a staining solution containing 100 μg/mL of RNase A and 40 μg/mL of propidium iodide (PI) in PBS for 20 min in the dark. Subsequently, samples were analysed on a BD FACSCanto II system (BD Biosciences, Le Pont de Claix, France) and PI incorporation estimated using the BD FACSDiva software (BD Biosciences, Le Pont de Claix, France).

### Iron dosage

Iron dosage was performed with the Iron Assay kit (Sigma Aldrich) according to the manufacturer’s instructions.

### Western blot analysis

Total proteins were isolated from GBM cells by sonication in a lysis buffer composed of 50 mM HEPES, pH 7.5, 150 mM NaCl, 1 mM EDTA, pH 8, 2.5 mM EGTA, pH 7.4, 0.1 % Tween 20, 10 % glycerol, 0.1 mM sodium orthovanadate, 1 mM sodium fluoride, 10 mM glycerophosphate and 0.1 mM phenylmethylsulfonyl fluoride (PMSF). Proteins (20 μg) were resolved on 4–20 % Mini-PROTEAN® TGX™ precast polyacrylamide gels (Bio-rad, Marnes-la-Coquette, France) and transferred to an Amersham GE Healthcare nitrocellulose membrane (0.45 μm pore size) (Fisher scientific, Illkirch, France). The following antibodies were used: a mouse anti-human Hypoxia-Inducible Factor-1α (HIF-1α) (610958, clone 54) (BD Biosciences, Le Pont De Claix, France) and an anti-human Mouse Heat Shock Cognate Protein 70 (HSC70) (sc-7298, B-6) (Santa Cruz Biotechnology, Heidelberg, Germany) was used as a loading control. These antibodies were diluted at a ratio of respectively 1:1000 and 1:10000, according to the manufacturer’s instructions. Goat anti-Mouse IgG Secondary Antibody, HRP conjugate (Fisher scientific, Illkirch, France) was used at a dilution of 1:2000. Detection was performed on SuperSignal™ West Femto Maximum Sensitivity Substrate (Fisher scientific, Illkirch, France) with a ChemiCapt 3000 imaging system (Vilber Lourmat, Marne-la-Vallée France).

### Statistical analysis

Three independent biological replicates were performed of all the experiments described in this manuscript. Statistical analyses were performed with R software using one- or two-way analysis of variance (ANOVA). Differences were considered significant at a *p*-value ≤0.05.

## Results and discussion

**Fig. 1:**
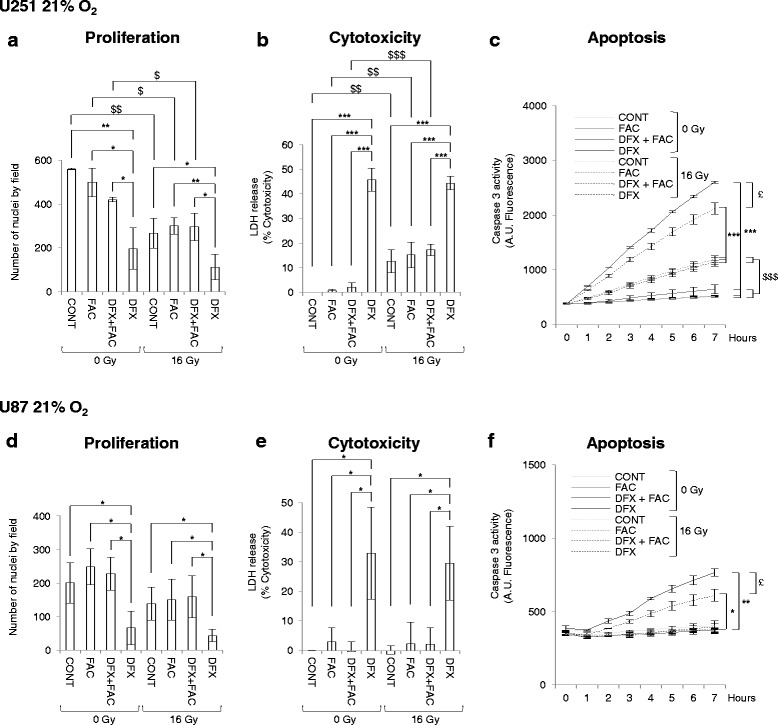
Deferasirox inhibits proliferation linked with increased cytotoxicity and apoptosis in glioblastoma cells under normoxic conditions. Number of nuclei of U251 (**a**) and U87 (**d**) glioblastoma cells cultivated at 21 % of oxygen in non-treated condition (CONT) or 3 days after treatment with 5 μM of ferric ammonium citrate (FAC), or with 10 μM of deferasirox and 5 μM of FAC (DFX + FAC) or with 10 μM of deferasirox (DFX) in non-irradiated condition (0 Gy) or following irradiation with 16 Gy (16 Gy). The number of nuclei are expressed as mean ± standard deviation (S.D.) (*n* = 3). Measure of lactate dehydrogenase (LDH) release into cell culture medium of U251 (**b**) and U87 (**e**) glioblastoma cells cultivated at 21 % of oxygen in untreated condition (CONT) or 3 days after treatment with 5 μM of ferric ammonium citrate (FAC), or with 10 μM of deferasirox and 5 μM of FAC (DFX + FAC) or with 10 μM of deferasirox (DFX) in non-irradiated condition (0 Gy) or following irradiation with 16 Gy (16 Gy). Cytotoxicity is expressed as mean percentage ± standard deviation (S.D.) (*n* = 3) of the total amount of LDH released from cells and relative to glioblastoma cells treated with 0.1 % Triton X-100, given the arbitrary percentage of 100. DEVD-AMC caspase 3 activity in U251 (**c**) and U87 (**f**) glioblastoma cells cultivated at 21 % of oxygen in untreated condition (CONT) or 3 days after treatment with 5 μM of ferric ammonium citrate (FAC), or with 10 μM of deferasirox and 5 μM of FAC (DFX + FAC) or with 10 μM of deferasirox (DFX) in non-irradiated condition (0 Gy) or following irradiation with 16 Gy (16 Gy). Caspase 3 activity is expressed as mean arbitrary units (A.U.) of fluorescence per 30 μg of proteins ± standard error of the mean (SEM) (*n* = 3). One-way ANOVA was performed between DFX treatment and CONT, FAC or DFX + FAC conditions in non-irradiated (0 Gy) or irradiated (16 Gy) conditions (^*^, *p*-value ≤0.05; ^**^, *p*-value ≤0.01; ^***^, *p*-value ≤0.001). Two-way ANOVA was performed between non-irradiated (0 Gy) condition and irradiated (16 Gy) condition (^$^, *p*-value ≤0.05; ^$$^, *p*-value ≤0.01; ^$$$^, *p*-value ≤0.001). Two-way ANOVA was performed between in DFX treatment in non-irradiated condition (0 Gy) and irradiated (16 Gy) condition (^£^, *p*-value ≤0.05)

Under experimental normoxic conditions, treatment with 10 μM of DFX for 3 days significantly inhibited proliferation of both U251 (Fig. 1a) and U87 (Fig. 1d) cells in comparison to control cells. The repression of proliferation was linked to high LDH release (Fig. 1b and e) and high caspase 3 activity (Fig. 1c and f), suggesting that the antiproliferative effect of DFX is probably mediated by cell death, including necrosis and apoptosis. In addition, phase-contrast microscopy photography clearly showed damage to the cells (Additional file 1: Figure S1). However, despite the ability of DFX to repress the proliferation at the same rate in glioblastoma cells, it led to more LDH release and more caspase 3 activity in U251 cells than in U87 cells. Importantly, when ferric ammonium sulfate (FAC) as Fe3+ donor was simultaneously added to the cell culture medium with DFX in stoichiometric proportion (i.e. 5 μM since two molecules of the tridentate DFX chelate one molecule of Fe3+), the antineoplastic effect of DFX was completely abolished, and, used alone, FAC failed to modulate the markers. This result confirms that the antineoplastic effect of DFX can be ascribed to its ability to chelate iron, thus emphasizing that iron is a key molecule for biological and biochemical processes.

Since radiotherapy is the main therapy for GBM to date, the same treatment was performed in combination with external irradiation. Sixteen hours after the beginning of DFX treatment, external X-ray beam radiation was applied at a dose of 16 Gy. Three days after irradiation, a significant decrease in proliferation was observed in U251 cells (Fig. 1a). Moreover, the impact of irradiation on U251 cells was correlated with the induction of LDH release (Fig. 1b) and caspase 3 activity (Fig. 1c). However, importantly, irradiation had no significant effect on U87 cells (Fig. 1d and f). These results suggest that U251 cells are more radiosensitive than U87 cells. These observations are consistent with previous work and may be explained by the fact that U251 cells have less DNA damage repair activity of Ape1 than in U87 cells [13].

Upon irradiation, DFX conserved its intrinsic characteristic of an antineoplastic agent in both cell lines, i.e. a repressor of proliferation, through increased LDH release and caspase 3 activity, compared to control irradiated cells (Fig. 1). This result showed that irradiation did not impair the activity of DFX in vitro. Importantly, irradiation did not overload the intrinsic antineoplastic activity of DFX nor generate a synergistic effect with DFX. It is important to note that DFX led to more caspase 3 activity at 0 Gy than at 16 Gy, demonstrating the potent apoptotic activity of this molecule (Fig. 1c and f). In addition, iron supplementation upon irradiation did not modulate the response in terms of proliferation, toxicity and apoptosis in the two cell lines, showing that excess of exogenous iron did not affect cell sensitivity to X-rays (Fig. 1).

**Fig. 2:**
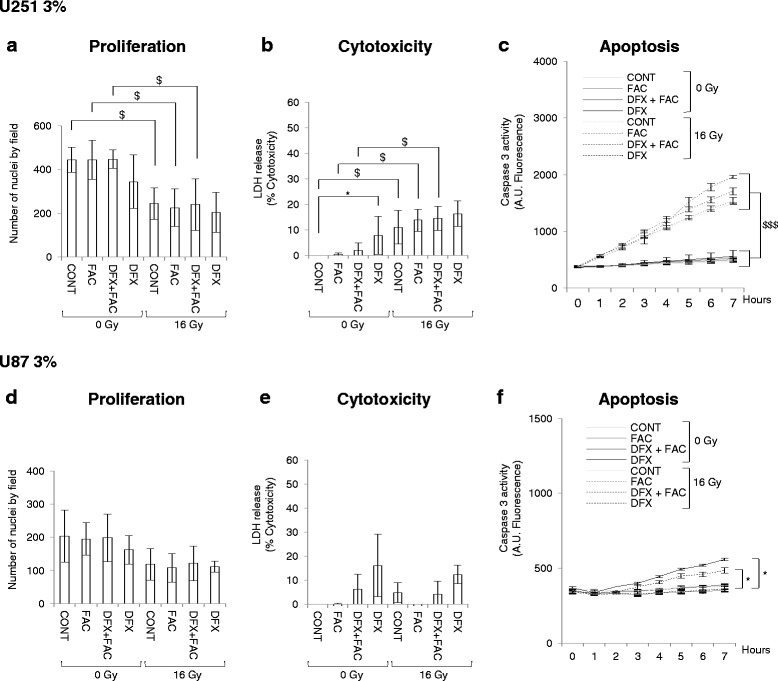
Antineoplastic effect of deferasirox is totally abolished in glioblastoma cells under mild-hypoxic conditions. The number of nuclei of U251 (**a**) and U87 (**d**) glioblastoma cells cultivated at 3 % of oxygen in untreated condition (CONT) or 3 days after treatment with 5 μM of ferric ammonium citrate (FAC), or with 10 μM of deferasirox and 5 μM of FAC (DFX + FAC) or with 10 μM of deferasirox (DFX) in non-irradiated condition (0 Gy) or following irradiation with 16 Gy (16 Gy). The numbers of nuclei are expressed as mean ± standard deviation (SD) (*n* = 3). Measure of lactate dehydrogenase (LDH) release into cell culture medium of U251 (**b**) and U87 (**e**) glioblastoma cells cultivated at 3 % of oxygen in untreated condition (CONT) or 3 days after treatment with 5 μM of ferric ammonium citrate (FAC), or with 10 μM of deferasirox and 5 μM of FAC (DFX + FAC) or with 10 μM of deferasirox (DFX) in non-irradiated condition (0 Gy) or following irradiation with 16 Gy (16 Gy). Cytotoxicity is expressed as mean percentage ± standard deviation (SD) (*n* = 3) of the total amount of LDH released from cells and relative to glioblastoma cells treated 0.1 % Triton X-100, given the arbitrary percentage of 100. DEVD-AMC caspase 3 activity in U251 (**c**) and U87 (**f**) glioblastoma cells cultivated at 3 % of oxygen in untreated condition (CONT) or 3 days after treatment with 5 μM of ferric ammonium citrate (FAC), or with 10 μM of deferasirox and 5 μM of FAC (DFX + FAC) or with 10 μM of deferasirox (DFX) in non-irradiated condition (0 Gy) or following irradiation with 16 Gy (16 Gy). Caspase 3 activity is expressed as mean arbitrary units (AU) of fluorescence per 30 μg of proteins ± standard rrror of the mean (SEM) (*n* = 3). One-way ANOVA was performed between DFX treatment and CONT, FAC or DFX + FAC conditions in non-irradiated (0 Gy) or irradiated (16 Gy) conditions (^*^, *p*-value ≤0.05). Two-way ANOVA was performed between non-irradiated (0 Gy) condition and irradiated (16 Gy) condition (^$^, *p*-value ≤0.05; ^$$$^, *p*-value ≤0.001)

As a hypoxic environment is frequently encountered in GBM due to the presence of areas of necrosis [14] and since the presence of hypoxic areas in GBM is correlated with the aggressive phenotype [15], we decided to perform the same treatment in a mild-hypoxic brain tumor environment. Surprisingly, the antiproliverative, cytotoxic and apoptotic effect of DFX was completely lost when cells from the two cell lines were cultivated in 3 % of oxygen (Fig. 2). Only the effect of the external irradiation at a dose of 16 Gy was conserved in U251 cells (Fig. 2a-c). To illustrate these results, no major cells damages were observed at 3 % of oxygen as shown in phase-contrast microscopy photography (Additional file 1: Figure S1). However, DFX led a slight significant induction of LDH for U251 cells (Fig. 2b) and caspase 3 activity for U87 cells in non-irradiated and irradiated conditions (Fig. 2f).

Importantly, same result was observed in human colon carcinoma, HCT116 cells in terms of proliferation where DFX treatment (10 μM for 3 days) led to inhibition of HCT116 proliferation under experimental normoxic conditions but was lost in in vitro hypoxic conditions (Additional file 2: Figure S2).

**Fig. 3:**
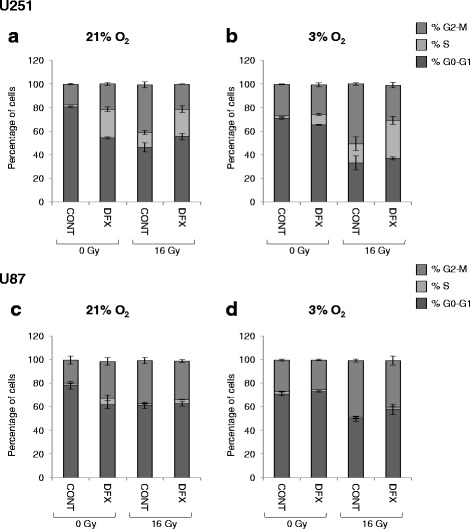
Deferasirox induced S and G2-M cell cycle arrest in glioblastoma cells but only under normoxic conditions. Cell cycle distribution in U251 glioblastoma cells cultivated at 21 % (**a**) or 3 % (**b**) of oxygen and in U87 glioblastoma cells cultivated at 21 % (**c**) or 3 % (**d**) of oxygen in untreated condition (CONT) or 3 days after treatment with 10 μM of deferasirox (DFX) in non-irradiated condition (0 Gy) or following irradiation with 16 Gy (16 Gy). Cell cycle distribution is expressed as percentage of cells in G0-G1, S and G2-M phases

A mechanism by which iron chelators exert their antineoplastic effects is by targeting iron dependant proteins that are key component in the progression of the cell cycle, such as ribonucleotide reductase [2], causing cell cycle arrest. Therefore, analysis of U251 and U87 cell cycles has been performed in absence or presence of DFX in combination or not with an irradiation scheme (Fig. 3 and Additional file 3: Figure S3). Under experimental normoxic conditions, treatment with 10 μM of DFX for 3 days induced an important accumulation of U251 cells in S phase and a slight accumulation in G2-M phase (Fig. 3a). Concerning U87 cells, 10 μM of DFX induced an important accumulation of cells in G2-M phase and a slight accumulation in S phases (Fig. 3c). This increase in the GBM cell number in S and G2-M phases was correlated to a diminution in the number of cells in G0-G1 phase (Fig. 3a and c). Importantly, the capacity of DFX to profoundly affect cell cycle distribution under experimental normoxic condition is lost at 3 % of oxygen (Fig. 3b and d) except for U251 cells where a slight accumulation in phase S is still observed at 3 % of oxygen (Fig. 3b).

Three days after irradiation, elevated accumulation of cells in the G2-M phase of the cell cycle is observed for both GBM cells and both conditions of oxygenation and accumulation in S phase is also observed but only for U251 cells (Fig. 3). Interestingly, although irradiation did not affect significantly U87 proliferation (Fig. 1), an accumulation in G2-M phase is observed (Fig. 3c and d).

Finally, when irradiation was combined to DFX, at 21 % of oxygen, cell cycle profiles are very similar to the one obtained with DFX alone (Fig. 3a and c and Additional file 3: Figure S3). In contrast, at 3 % of oxygen, the combination of irradiation with DFX resulted in a cell cycle profile rather similar to the one obtained with radiation alone, while DFX was even capable to slightly reduce the impact of radiations on cell accumulation in G2-M phase.

In this last condition, the increase in S phase, particularly for U251 cells, revealed an effect of the iron chelator that was initially hidden in the overall analysis of cell proliferation (Fig. 3b and d and Additional file 3: Figure S3). As such, DFX seems to mainly interfere with S-phase, which has already been related to the specific expression of the ribonucleotide reductase [2], requiring iron for its activity which catalyzes the rate limiting step in the production of deoxyribonucleotides needed for DNA synthesis. Meanwhile, irradiation leads more a G2-M blockade, important phase for DNA damage checkpoint upstream DNA repair or cell death. However, whatever their intrinsic impact, nearly abolish for DFX used alone at 3 %, DFX and irradiation did not presented any synergism nor on proliferation or on the cell cycle.

**Fig. 4:**
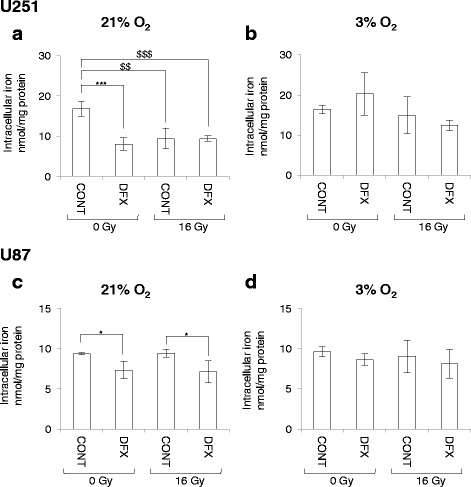
Intracellular iron concentration is depleted by both deferasirox and irradiation in U251 cells but only under normoxic condition. Intracellular iron concentration in U251 glioblastoma cells cultivated at 21 % (**a**) or 3 % (**b**) of oxygen and in U87 glioblastoma cells cultivated at 21 % (**c**) or 3 % (**d**) of oxygen in untreated condition (CONT) or 3 days after treatment with 10 μM of deferasirox (DFX) in non-irradiated condition (0 Gy) or following irradiation with 16 Gy (16 Gy). Intracellular iron concentration is expressed in nmol per mg of protein as mean ± standard deviation (SD) (*n* = 3). One-way ANOVA was performed between DFX treatment and CONT in non-irradiated (0 Gy) or irradiated (16 Gy) conditions (^*^, *p*-value ≤0.05; ^***^, *p*-value ≤0.001). Two-way ANOVA was performed between non-irradiated (0 Gy) condition and irradiated (16 Gy) condition (^$$$^, *p*-value ≤0.001)

To better understand why the effect of DFX is lost at 3 % of oxygen in vitro, iron dosage was performed. The iron contents measured in both GBM cells are in line with values found in primary rat astrocytes (9.3 ± 1.2 nmol per mg protein) [16] (Fig. 4). DFX treatment at 10 μM for 3 days in normoxic condition led to significant intracellular iron depletion in both cell lines (Fig. 4a and c). Moreover, irradiation significantly repressed intracellular iron contents but only in U251 cells in normoxic condition (Fig. 4a). Importantly, no repression of iron content by DFX was measured with 3 % O_2_ although there was no difference between the total concentration of iron in the two oxygenation conditions (Fig. 4b and d). This result suggests that the effect of DFX on intracellular iron depletion observed at 21 % of oxygen is absent at 3 % of oxygen. Since the antineoplastic effect of DFX is due to its ability to chelate iron, as FAC addition abolishes the toxicity of DFX, this could explain the absence of antineoplastic effect of DFX at 3 % of oxygen. As oxidation is less frequent in hypoxic condition than in normoxic condition [17], it is possible that at 3 % of oxygen, more Fe2+ is present than Fe3+. As DFX is selective for Fe3+ chelation [18], this hypothesis could explain the loss of DFX effect in mild-hypoxic condition. In order to confirm this hypothesis, an iron dosage to distinguish the ferrous (Fe2+) and ferric (Fe3+) forms should be used to get round the limit of detection of these two forms of iron.

The loss of antineoplastic activity of DFX in an in-vitro mild-hypoxia environment could raise the question of the benefits of using iron chelators in anticancer therapy where the tumor is often hypoxic [12]. The synthesis of iron chelators that efficient only in hypoxic cells, such as bioreductive prodrugs [17], might overcome this limitation. Another alternative would be the synthesis of iron chelators that are only active in cancer cells. This has already been achieved in neurodegenerative diseases where iron accumulation has been clearly linked to these diseases [4]. In Alzheimer’s disease, the finding that acetylcholinesterase (AChE) colocalizes with amyloid-β and accelerates its aggregation has led to the development of a new class of selective AChE inhibitors with site-activated chelating activity. The prochelator HLA20A exhibits low affinity for metal ions, but can be activated following AChE cleavage to liberate an active chelator and an AChE inhibitor [19]. The active molecule HLA20 possesses neuroprotective properties both in vitro and in vivo with the ability to inhibit β-amyloid aggregation induced by metal ions [19]. An additional strategy could be to take advantage of cancer cell metabolism to build a site-directed iron chelator. In this context, the synthesis of a new generation of iron chelators such as quilamines has produced encouraging results [20]. Quilamines are linked to linear polyamine vectors that use the polyamine transport system, which is overexpressed in most cancer cells [20].

**Fig. 5:**
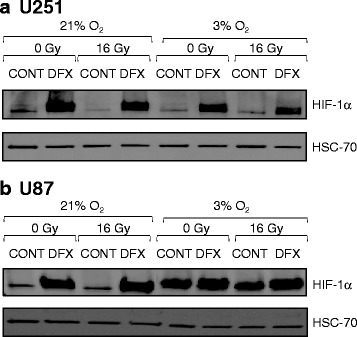
Deferasirox induced hypoxia-inducible factor -1α (HIF-1α) protein stabilization. Levels of hypoxia-inducible factor -1α (HIF-1α) protein and heat shock cognate protein 70 (HSC70) protein used as loading control protein in U251 glioblastoma cells cultivated at 21 % of oxygen or 3 % of oxygen (**a**) and in U87 glioblastoma cells cultivated at 21 % of oxygen or 3 % of oxygen (**b**) in untreated condition (CONT) or 3 days after treatment with 10 μM of deferasirox (DFX) in non-irradiated condition (0 Gy) or following irradiation with 16 Gy (16 Gy). Western blot data represent one of three independent experiments with comparable results

Another dilemma linked with iron chelators is that iron depletion may inhibit prolyl hydroxylase domain (PHD) enzyme activity and therefore activate the hypoxia-inducible factor-1 (HIF-1) pathway, which is implicated in tumor aggressiveness and invasion [21]. Indeed, in both GBM cells, HIF-1α protein stabilization has been observed following DFX treatment in normoxic condition (Fig. 5). Importantly, the mild-hypoxic condition led to stabilization of HIF-1α in U87 cells (Fig. 5b) but not in U251 cells (Fig. 5a), suggesting that in this GBM cell line, an oxygen concentration of 3 % is not low enough to observe intracellular accumulation of HIF-1α. However, at this low level of oxygen, DFX was still able to stabilize the HIF-1α protein in U251 cells and the same trend was observed in U87 despite the fact that the stabilization was largely masked by the high level of HIF-1α in these low oxygen conditions. Moreover, irradiation did not significantly impact HIF-1α expression or DFX-mediated HIF-1α stabilization in either type of cell. Finally, HIF-1α protein stabilization mediated by DFX observed in all the conditions tested (Fig. 5), suggests that the difference in the antineoplastic effect by DFX observed at 21 and 3 % of oxygen should not be attributed to HIF-1 signaling. The consequence of this HIF-1α stabilization could be dramatic for anticancer therapy. Indeed, it has been shown that iron depletion by desferrioxamine (DFO), which leads to HIF-1 activation and expression of urokinase plasminogen activator (uPAR) and matrix metallopeptidase 2 (MMP2), enhanced invasion of GBM cells by degrading the extracellular matrix (ECM) [22]. In addition, in clinical trials with DFO, toxicities including edema were reported [23], probably due to its ability to increase the potent angiogenic factor vascular endothelial growth factor (VEGF) [24], a well characterized HIF-1 target gene. Taken together, these data underline the need for caution when using chelators in cancer therapy, particularly for tumors with high invasive potential.

Moreover, it is still difficult to predict if iron chelation is able to affect all the GBM tumor margins and how homogeneously tumor mass rapid dividing cells and glioblastoma stem cells (GSC) would respond to iron chelation. Concerning GSC, chelation therapy may have an impact on cancer stem cells since it has been recently shown that iron dependency is enhanced in GSC [25]. However, it is important to notice that GBM tumors are composed of GSC plastic cells (defined as proliferative, symmetrically dividing and less invasive cells) and GSC rigid cells (defined as quiescent, asymmetrically dividing and more invasive cells) [26]. Since iron chelators preferentially target cells with high proliferative capacity, DFX might not be selective for GSC rigid cells responsible for tumor recurrence. Taken together, impact of DFX on GBM cells and GSC warrants further investigation.

Another limit to using iron chelators in brain tumors is the difficulty in crossing the blood–brain barrier (BBB). In neurodegenerative diseases, Novartis claims improved penetration of DFX into the brain through the co-administration of an efflux protein inhibitor in the patent US20090306160A1. Among other alternatives, drug delivery nanosystems derived from nanotechnologies are perhaps the most appropriate and potentially the most useful in this biological context [27]. Drugs encapsulated in nanoparticles may be more soluble, and have improved biological barrier crossing properties and better controlled release kinetics, with substantial clinical advantages including dose reduction, prevention of side effects and improvement of bioavailability within the targeted tumor cells [27]. Such nano-objects can either be implanted inside the tumor or within the resection cavity or, alternatively, delivered via the blood to the CNS tumor site. Brain locoregional active targeting by direct infusion by convection-enhanced delivery (CED) into the brain could lead to a major breakthrough in efficacy while allowing optimum specificity and safety [28].

Concerning iron chelation therapies, some studies of nano-carriers are currently being conducted for use in neurodegenerative diseases [29–31]. Conjugating a derivative of deferiprone with nanoparticles did not alter its ability to chelate iron. This nano-deferiprone analog conjugate was shown to be able to inhibit amyloid-β aggregation in vitro and to protect neuronal cells from amyloid-β-associated neurotoxicity [30, 31]. DFX has been conjugated to lactoferrin, which was able to cross the BBB via its receptors. The neuroprotective effects of this nano-object have been assessed in vitro and in vivo. The results revealed a significant reduction in learning deficits induced by amyloid-β injection in a rat model of Alzheimer’s disease [29]. However, none of these objects have yet been tested in the context of brain tumors, including in GBM, and locoregional application should probably be improved. 

## Conclusions

Taken together, the results of the present work underline the fact that iron depletion by iron chelators and their application in anticancer strategies is much more complex than initially thought. Since DFX does not synergize with irradiation and as low oxygen tension reverses its activity in vitro, developing new chemistry or a drug delivery system that would keep DFX active in hypoxic cells should be the next step in its clinical development.

## Additional files

Additional file 1: Figure S1. Phase-contrast microscopy photography of U251 and U87 glioblastoma cells cultivated at 21 % or 3 % of oxygen in non-treated condition (CONT) or 3 days after treatment with 5 μM of ferric ammonium citrate (FAC), or with 10 μM of deferasirox and 5 μM of FAC (DFX + FAC) or with 10 μM of deferasirox (DFX) in non-irradiated condition. Original magnification 40 ×. (PDF 145 kb) Additional file 2: Figure S2. Number of nuclei of HCT116 human colon carcinoma cells cultivated at 21 % or 3 % of oxygen in non-treated condition (CONT) or 3 days after treatment with 10 μM of deferasirox (DFX) in non-irradiated condition. The number of nuclei are expressed as mean ± standard deviation (S.D.). One-way ANOVA was performed between DFX treatment and CONT in the two conditions of oxygenation (**, *p*-value ≤0.01). (PDF 52 kb) Additional file 3: Figure S3. Representative cell cycle profiles for U251 and U87 glioblastoma cells cultivated at 21 % or 3 % of oxygen in untreated condition (CONT) or 3 days after treatment with 10 μM of deferasirox (DFX) in non-irradiated condition (0 Gy) or following irradiation with 16 Gy (16 Gy). (PDF 103 kb)
