# Supplementary material for: Low oxygen tension reverses antineoplastic effect of iron chelator deferasirox in human glioblastoma cells
Source: BMC Cancer. 2016 Feb 1;16:51. doi: 10.1186/s12885-016-2074-y (PMC4736662; doi:10.1186/s12885-016-2074-y)
Supplement: Additional file 1: Figure S1. — Phase-contrast microscopy photography of U251 and U87 glioblastoma cells cultivated at 21 % or 3 % of oxygen in non-treated condition (CONT) or 3 days after treatment with 5 μM of ferric ammonium citrate (FAC), or with 10 μM of deferasirox and 5 μM of FAC (DFX + FAC) or with 10 μM of deferasirox (DFX) in non-irradiated condition. Original magnification 40 ×. (PDF 145 kb) [file 12885_2016_2074_MOESM1_ESM.pdf]

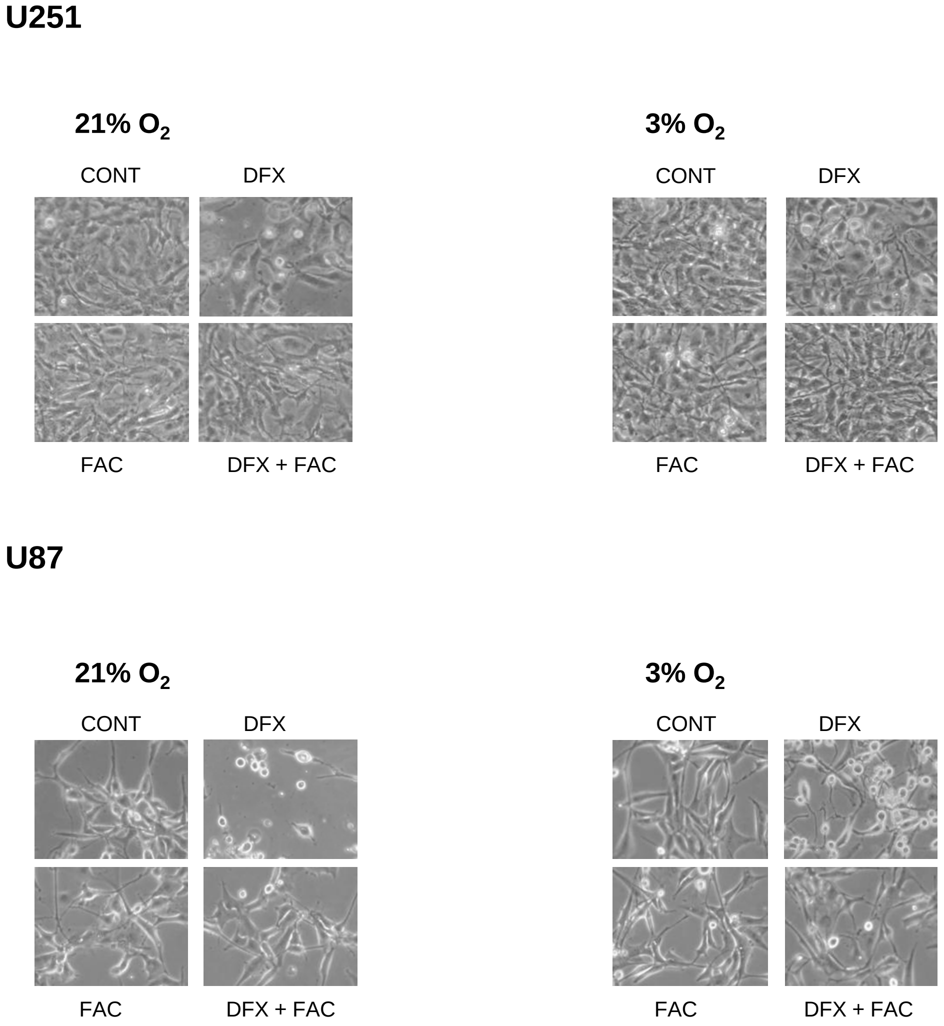

Figure S1.
Phase-contrast microscopy photography of U251 and U87 glioblastoma cells cultivated at 21% or 3% of oxygen in non-treated condition (CONT) or 3 days after treatment with 5 µM of ferric ammonium citrate (FAC), or with 10 µM of deferasirox and 5 µM of FAC (DFX + FAC) or with 10 µM of deferasirox (DFX) in non-irradiated condition. Original magnification 40 x.
