# Supplementary material for: Low oxygen tension reverses antineoplastic effect of iron chelator deferasirox in human glioblastoma cells
Source: BMC Cancer. 2016 Feb 1;16:51. doi: 10.1186/s12885-016-2074-y (PMC4736662; doi:10.1186/s12885-016-2074-y)
Supplement: Additional file 2: Figure S2. — Number of nuclei of HCT116 human colon carcinoma cells cultivated at 21 % or 3 % of oxygen in non-treated condition (CONT) or 3 days after treatment with 10 μM of deferasirox (DFX) in non-irradiated condition. The number of nuclei are expressed as mean ± standard deviation (S.D.). One-way ANOVA was performed between DFX treatment and CONT in the two conditions of oxygenation (**, p-value ≤0.01). (PDF 52 kb) [file 12885_2016_2074_MOESM2_ESM.pdf]

HCT116 cells

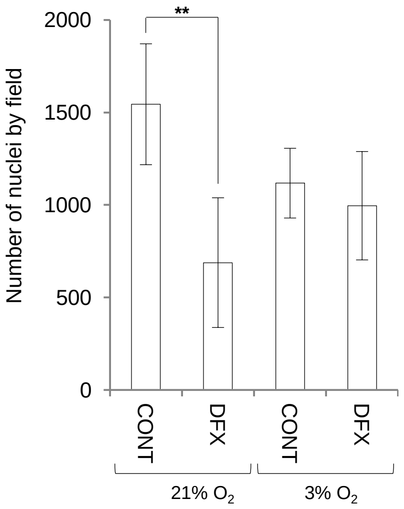

Figure S2.

Number of nuclei of HCT116 human colon carcinoma cells cultivated at 21% or 3% of oxygen in non-treated condition (CONT) or 3 days after treatment with 10 µM of deferasirox (DFX) in non-irradiated condition. The number of nuclei are expressed as mean ± standard deviation (S.D.). One-way ANOVA was performed between DFX treatment and CONT in the two conditions of oxygenation (**, $p\text{-value} \leq 0.01$).
