# Supplementary material for: Low oxygen tension reverses antineoplastic effect of iron chelator deferasirox in human glioblastoma cells
Source: BMC Cancer. 2016 Feb 1;16:51. doi: 10.1186/s12885-016-2074-y (PMC4736662; doi:10.1186/s12885-016-2074-y)
Supplement: Additional file 3: Figure S3. — Representative cell cycle profiles for U251 and U87 glioblastoma cells cultivated at 21 % or 3 % of oxygen in untreated condition (CONT) or 3 days after treatment with 10 μM of deferasirox (DFX) in non-irradiated condition (0 Gy) or following irradiation with 16 Gy (16 Gy). (PDF 103 kb) [file 12885_2016_2074_MOESM3_ESM.pdf]

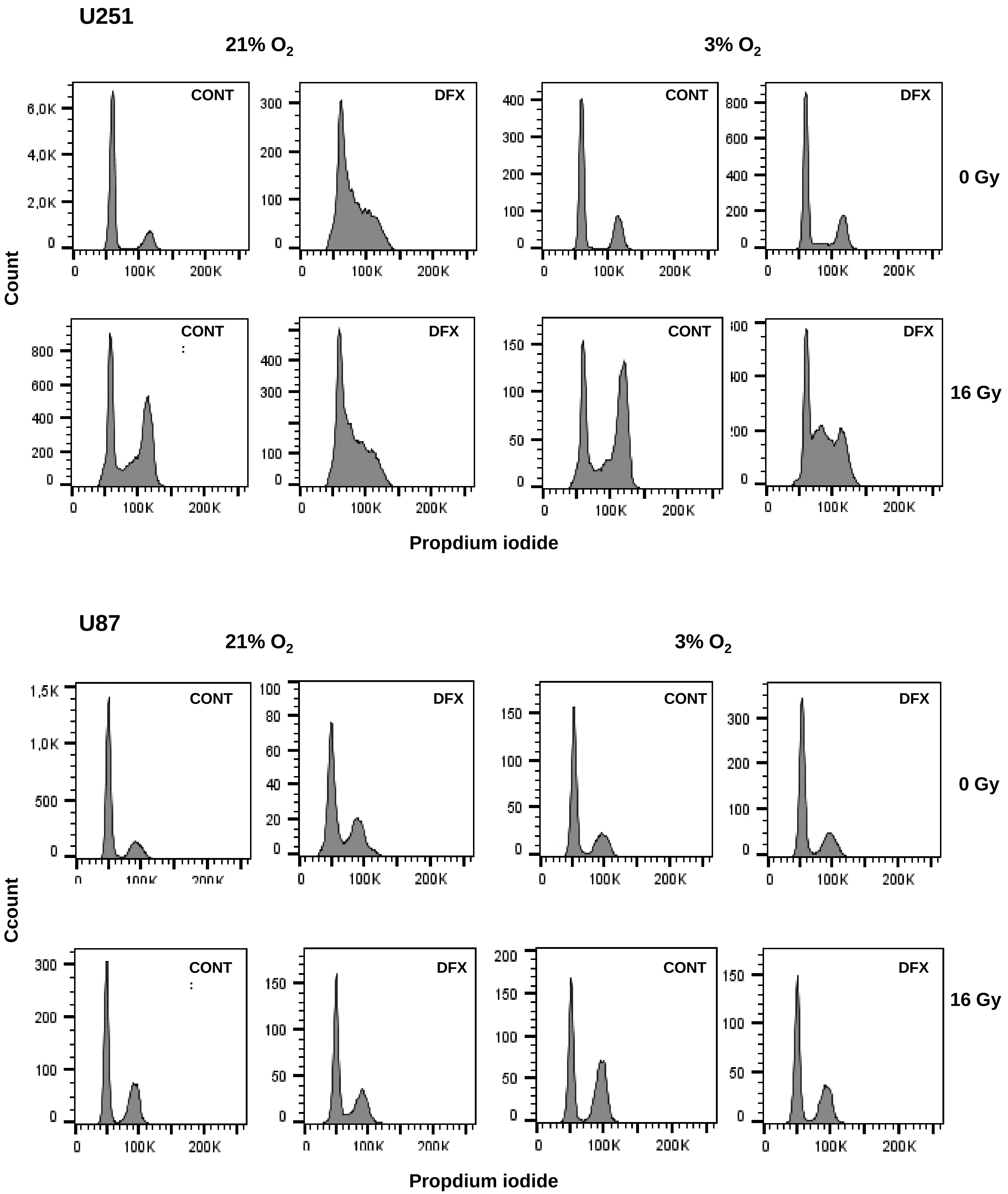

Figure S3.

Representative cell cycle profiles for U251 and U87 glioblastoma cells cultivated at 21% or 3% of oxygen in untreated condition (CONT) or 3 days after treatment with 10 µM of deferasirox (DFX) in non-irradiated condition (0 Gy) or following irradiation with 16 grays (16 Gy).
